# Preparation of Cu-Containing Substances via an Ultrasonic-Assisted Solvothermal Approach and Their Catalytic Effects on the Thermal Decomposition of Ammonium Perchlorate

**DOI:** 10.3390/ma18132928

**Published:** 2025-06-20

**Authors:** Cheng-Hsiung Peng, Pin-Hsien Su, Jin-Shuh Li, Yan-Jun Ke

**Affiliations:** 1 Department of Applied Materials Science and Technology, Minghsin University of Science and Technology, Hsinchu 304, Taiwan; c12180011@std.must.edu.tw; 2 Department of Chemical and Materials Engineering, Chung Cheng Institute of Technology, National Defense University, Taoyuan 335, Taiwan; samuel6142851@gmail.com (P.-H.S.); lijinshuh@ccit.ndu.edu.tw (J.-S.L.)

**Keywords:** ultrasonic-assisted method, solvothermal, copper benzene-1,3,5-tricarboxylate, ammonium perchlorate, catalysis

## Abstract

In this study, a one-pot, ultrasonic-assisted solvothermal method was successfully employed to prepare three copper-containing compounds: copper benzene-1,3,5-tricarboxylate (Cu_3_(BTC)_2_), copper powder, and copper-metalized activated carbon (Cu@AC). This method is efficient and safe and has potential for use in scalable production. The characteristics of the resulting products were analyzed using various techniques, including X-ray diffraction (XRD), scanning electron microscopy (SEM), specific surface area measurement along with pore size distribution, and thermogravimetric analysis–differential scanning calorimetry (TG-DSC). Additionally, the catalytic effects of these products on the thermal decomposition of ammonium perchlorate (AP) were evaluated. All three substances were found to lower the thermal decomposition temperature of AP and enhance heat release. Cu_3_(BTC)_2_ demonstrated exceptional catalytic performance and compatibility with AP, as shown using the vacuum stability test (VST). The thermal analysis results indicated that the thermal decomposition temperature and apparent activation energy of AP decreased from ~442 °C to around 340 °C and from ~207 kJ mol^−1^ to approximately 128 kJ mol^−1^, respectively, when 3 wt% Cu_3_(BTC)_2_ was contained in AP. Moreover, the heat released via the exothermic decomposition of AP increased from 740 J g^−1^ to1716 J g^−1^. A possible reaction mechanism is proposed based on the evolved gas analysis (EGA) findings to explain the observed catalytic effects.

## 1. Introduction

Ammonium perchlorate (NH_4_ClO_4_, AP) is the most commonly used oxidizer and high-energy component in solid composite propellants and polymer-bonded explosives (PBXs) [1,2,3,4,5]. Its widespread use is attributed to its high oxygen content and exothermic decomposition properties. Several studies [5,6,7,8,9,10,11,12,13] have shown that introducing a catalyst can lower the activation energy required for the pyrolysis of AP, alter its reaction mechanism, and thus change the composition of the final products. These modifications can increase the reaction rate and the heat released during decomposition, which is crucial in enhancing the performance of solid propellants and the explosive power of warhead charges. Consequently, this area of research is a priority in the development of military technology.

The thermal decomposition catalysts used for AP mainly consist of various transition metal-containing materials. These include elemental substances, including nanoparticles composed of nickel [14], copper [15], and zinc [16] alloys, such as Al-Cu-Ni particles [17] and Mn-based bi-metallic nanocomposites [18], and metallic oxides, including α-Fe_2_O_3_ [19], Cu_2_O [20], and CuCr_2_O_4_ [21]. Recently, transition metal-organic frameworks (MOFs) such as ZIF-67 [22], Fe-Co MOF [23], MIL-88B [24], energetic organometallic compounds [25], including [Cu(5-amino-1H-tetrazole)_4_]Cl_2_ [26] and Cu(4,4′-azo-1,2,4-triazole)_3_(NO_3_)_2_ [27], and advanced metalized carbon materials, such as copper–graphene oxide nanocomposites [28,29,30,31], have also been used in this context. These catalysts typically make up about 2–5 wt% of AP and are effective in lowering the thermal decomposition temperature of AP and enhancing the heat release. Although these catalysts exhibit considerable diversity and unique morphological characteristics, it is important to note that while some materials are innovative, others may pose safety risks. In addition, obtaining the starting materials can be expensive, and the preparation processes may be complex, time-consuming, or inherently hazardous. For example, some energetic organometallic compounds contain explosophoric groups, which may be hazardous and cause accidents [25,26,27]. Advanced lightweight carbon materials, including carbon nanotubes and graphene, present challenges with regard to mass production [28,29,30,31] and may result in non-homogeneous mixing when incorporated into high-energy material formulations. It is especially important to recognize that every advantage comes with a corresponding disadvantage. Although these catalysts demonstrate the potential to lower the initiation temperature of AP pyrolysis and facilitate exothermic reactions, concerns may arise regarding their compatibility with AP, which could affect the thermal stability of the resulting products. Therefore, a comprehensive evaluation of the synergistic effects of these catalysts is essential, considering factors such as their economic feasibility, engineering applicability, and process safety.

Extensive research has shown that copper-containing substances possess exceptional catalytic properties. These compounds are effective in promoting the thermal decomposition of AP and are valuable across various fields [15,20,26,31], including chemical engineering [32], photocatalysis [33], and biomedicine [34]. Given their dual-use potential in both military and civilian applications, this study aims to develop a versatile process that is both convenient and efficient in terms of time and energy for synthesizing three copper-containing substances: copper benzene-1,3,5-tricarboxylate (Cu_3_(BTC)_2_), copper powder, and copper-metalized activated carbon (Cu@AC).

Conventional wet-chemical methods typically require high temperatures and lengthy reaction times for the synthesis and crystallization processes. For instance, Al-Janabi et al. reported an optimized procedure for synthesizing Cu_3_(BTC)_2_ in a sealed autoclave at 100 °C for 24 h [35]. Fievet et al. synthesized fine copper particles using a polyol process at temperatures ranging from 175 °C to 195 °C, with reaction times varying from 30 min to 2 h, which required stirring and refluxing [36]. Li et al. prepared Cu/graphene oxide nanocomposites through a series of processes, including solvothermal treatment at 180 °C for 4 h, freeze-drying at −80 °C for 5 h, and then calcination at 500 °C for 2 h under Ar [31].

Recently, sonochemical methods have gained recognition in organic synthesis for their effectiveness in improving mixing homogeneity and accelerating reaction and crystallization rates [37,38,39]. These methods offer greater convenience and control compared to traditional techniques, allowing many organic reactions to be performed under ultrasonic irradiation with high yields in a shorter timeframe. For instance, Li et al. demonstrated an efficient and environmentally friendly approach to producing submicron Cu_3_(BTC)_2_ using an ultrasonic method, which dramatically shortens reaction times to less than 60 min at ambient temperature and pressure [40].

In light of this, we implemented a one-pot ultrasonic-assisted solvothermal method to synthesize the three copper-containing substances. Among them, Cu_3_(BTC)_2_ was synthesized through a chelation reaction between Cu^2+^ ions and BTC^3−^ organic ligands [40,41], while copper powder and Cu@AC were produced via the reduction of Cu^2+^ ions using ethylene glycol [36,42,43]. This method utilized readily available chemicals, aiming to achieve a facile, generalizable, cost-effective, and environmentally sustainable approach.

We evaluated the properties of the obtained products and confirmed that all three copper-containing substances effectively catalyzed the thermal decomposition of AP. Notably, Cu_3_(BTC)_2_ exhibited the best catalytic effect among them. Given the lack of the existing literature on the use of Cu_3_(BTC)_2_ as a catalyst for the thermal decomposition of AP, this study further evaluated its catalytic potential. We determined the apparent activation energy (E_a_) and monitored the reaction progress during the pyrolysis of AP through thermal analysis combined with evolved gas analysis (EGA). Based on the experimental results, we propose a possible reaction mechanism. Additionally, standard testing demonstrated a favorable compatibility between Cu_3_(BTC)_2_ and AP.

## 2. Materials and Methods

### 2.1. Raw Materials

All the chemicals used in this study were reagent-grade and used without further purification. The following were obtained from Sigma-Aldrich (Darmstadt, Germany): copper (II) nitrate trihydrate [Cu(NO_3_)_2_·3H_2_O, 99%], benzene-1,3,5-tricarboxylic acid (H_3_BTC, 95%), ammonium perchlorate (NH_4_ClO_4_, 99.5%), dimethylformamide (DMF, 99%), activate carbon (AC, amorphous, −325 mesh, 99%, specific surface area ~500 m^2^ g^−1^), and sodium hydroxide (NaOH, 99%). Ethylene glycol (EG, 99%) and absolute ethanol (EtOH) were sourced from Thermo Scientific Chemicals (Waltham, MA, USA).

### 2.2. Preparation of Cu-Containing Substances

#### 2.2.1. Preparation of Cu_3_(BTC)_2_

Firstly, 5 mmol (1.05 g) of H_3_BTC was dissolved in a mixture of 60 mL of absolute ethanol and 40 mL of DMF. The mixture was stirred for 10 min until it became clear. Next, 7.5 mmol of Cu(NO_3_)_2_·3H_2_O (1.81 g) was added, and the solution was stirred for an additional 10 min until fully dissolved. The resulting blue solution was then transferred into a 250 mL Teflon container and subjected to a solvothermal reaction in an ultrasonic autoclave reactor (Parr 4760, Parr Instrument Company, Moline, IL, USA), customized with programmable logic ultrasonic and temperature controllers) at 80 °C for 2 h. During this process, ultrasonic assistance was applied simultaneously at a frequency of 20 kHz and 400 W. After the reaction and subsequent cooling to room temperature, a blue powder was collected through centrifugation, filtration, and washing with anhydrous ethanol. Finally, the powder was activated under vacuum at 120 °C for 12 h, yielding the target product, Cu_3_(BTC)_2_, which appeared violet. The product yield was calculated as the ratio of the mass of copper in the resulting Cu_3_(BTC)_2_ to the mass of copper in the Cu(NO_3_)_2_·3H_2_O reactant used. The detailed synthesis procedure is provided in Figure S1 of the Supplementary Materials.

#### 2.2.2. Preparation of Cu Powder

A total of 10 mmol of Cu(NO_3_)_2_·3H_2_O (2.42 g) was dissolved in EG to form a 0.10 M solution and then thoroughly mixed with 1.0 M NaOH in an EG solution with a Cu^2+^-to-OH^−^ molar ratio of 1:3. The resultant sol underwent an ultrasonic-assisted solvothermal process at 20 kHz and 400 W at 120 °C for 1 h. After the EG reduction reaction, the solid in the suspension was collected via centrifugation and filtration, washed with anhydrous ethanol, and vacuum-dried, yielding a reddish-brown product. The yield of the product was calculated as the ratio of the mass of Cu powder to the mass of copper contained in the reactant, Cu(NO_3_)_2_·3H_2_O. The detailed synthesis procedure is provided in Figure S2 of the Supplementary Materials.

#### 2.2.3. Preparation of Cu@AC

In a 250 mL Teflon container, 2 g of AC was mixed with 100 mL of EG and sonicated for homogenization. Then, 100 mL of 0.01 M Cu(NO_3_)_2_·3H_2_O in EG was added and stirred for 1 h. A 1.0 M NaOH/EG solution was then added dropwise to achieve a Cu^2+^-to-OH^−^ molar ratio of 1:3. The mixture underwent the ultrasonic-assisted solvothermal process at 20 kHz and 400 W for 1 h at 120 °C. Afterward, the solid was separated via centrifugation and filtration, washed with anhydrous ethanol, and vacuum-dried, resulting in a black and brownish-yellow product. The copper content in the Cu@AC product was determined by dissolving the copper with dilute nitric acid to form Cu^2+^ ions, whose concentration was then measured via ethylenediaminetetraacetic acid (EDTA) titration, allowing for the calculation of the copper content. The detailed synthesis procedure is provided in Figure S3 of the Supplementary Materials.

### 2.3. Characterization

Powder X-ray diffraction (XRD) patterns were recorded in the 2θ range of 5° to 80° using a Bruker D8 A25 diffractometer (Billerica, MA, USA) with Cu Kα radiation. The morphological features and elemental composition of the obtained products were examined using a scanning transmission electron microscope (STEM, JEM-2100, JOEL, Tokyo, Japan), which was equipped with energy-dispersive X-ray spectroscopy (EDS, JED-2000, Tokyo, Japan). The specific surface area (S_BET_) and pore volume (V_p_) of the tested samples were measured using the single-point Brunauer–Emmett–Teller (BET) nitrogen adsorption method (Micromeritics TriStar II plus, Norcross, GA, USA). Pore size distribution was evaluated using the Barrett–Joyner–Halen (BJH) method.

A specific amount of AP and each prepared copper-containing substance were placed into an agate mortar, maintaining a fixed weight ratio of the prepared copper-containing substances to AP at 3:97. The mixture was then thoroughly ground until it reached a uniform consistency, and test samples were obtained. To evaluate the catalytic effects of the resulting copper-containing substances, simultaneous analysis was conducted using thermogravimetry (TG) and differential scanning calorimetry (DSC). This analysis was performed from ambient temperature to 500 °C using a thermal analyzer (Netzsch, STA 409, Bavaria, Germany). The apparent activation energy (E_a_) of the thermal decomposition reaction was determined using the Kissinger and Ozawa methods, based on the DSC data obtained from heating the test sample at four different rates: 1 °C min^−1^, 2 °C min^−1^, 5 °C min^−1^, and 10 °C min^−1^, as detailed in our previous study [44]. Additionally, evolved gas analysis (EGA) was performed by examining the gaseous species released from the TG-DSC cell, utilizing a mass spectrometer (Netzsch, QMS 403D, Bavaria, Germany).

The compatibility between the prepared Cu3(BTC)2 and AP was evaluated using a vacuum stability test (VST) in accordance with MIL-STD-1751A, method 1063 [45]. This methodology is the same as that used in our previous research [46]. A sample weighing 5 g was placed in a glass tube and sealed with a stopper that included pressure and temperature transducers. The gas generated by the thermal decomposition of the test sample was measured under vacuum at 100 °C for 40 h. According to MIL-STD-1751A, the maximum allowable gas release should not exceed 2 mL g^−1^.

## 3. Results and Discussion

### 3.1. Characterization of the Resultant Products

**Figure 1:**
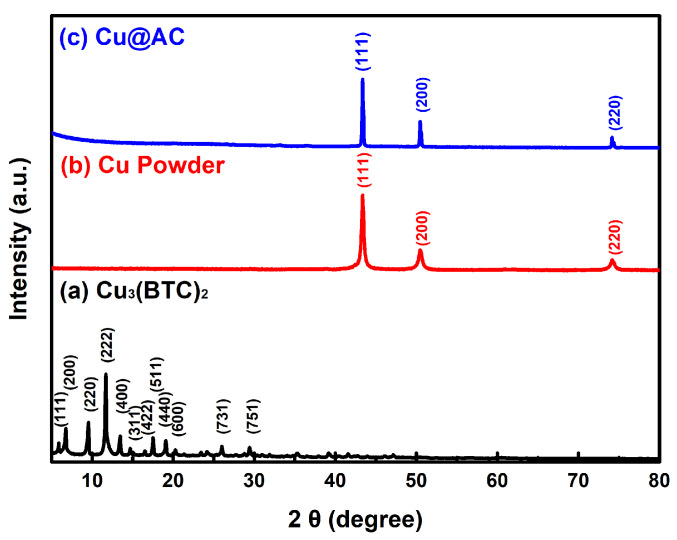
XRD patterns of the as-prepared products: (**a**) Cu_3_(BTC)_2_, (**b**) Cu powder, and (**c**) Cu@AC.

Figure 1 shows the XRD patterns of three products prepared using the ultrasonic-assisted solvothermal approach. In Figure 1a, the characteristic peaks at 2θ = 6.5°, 9.5°, 11.5°, and 13.4° correspond to the crystalline structure of Cu_3_(BTC)_2_, which aligns with the available data in the literature [47,48] of the JCPDS file 23-00380. No impurities were detected, confirming the successful formation of high-purity Cu_3_(BTC)_2_. In Figure 1b,c, the diffraction peaks observed at 2θ = 43.3°, 50.5°, and 74.1° correspond to the (111), (200), and (220) crystal planes of the face-centered cubic copper, respectively, which match the JCPDS file 04-0836. The absence of additional peaks associated with impurities suggests that only metallic Cu was formed, without oxides such as Cu_2_O or CuO.

**Figure 2:**
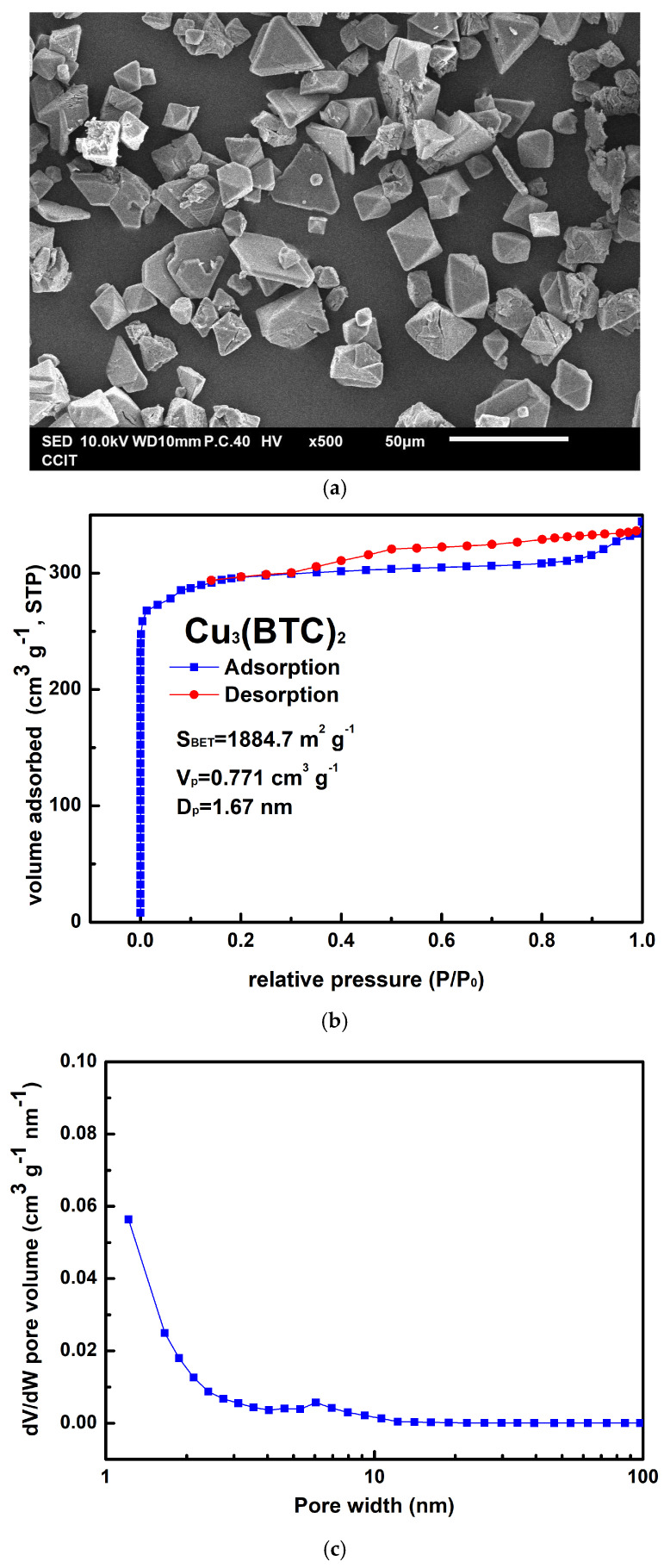
(**a**) Typical SEM image of Cu_3_(BTC)_2_, (**b**) nitrogen adsorption–desorption isotherm of Cu_3_(BTC)_2_, and (**c**) pore size distribution curve of Cu_3_(BTC)_2_.

Figure 2a shows the as-prepared Cu_3_(BTC)_2_ crystals, which could be either granular or octahedral in shape, with sizes ranging from 10 µm to 30 µm. The preparation process resulted in an approximately 82% yield for Cu_3_(BTC)_2_ based on the initial amount of Cu^2+^ used. In Figure 2b, the Cu_3_(BTC)_2_ sample demonstrates a typical Type I isotherm, characterized by an H_4_ hysteresis loop, as defined by the International Union of Pure and Applied Chemistry (IUPAC). This isotherm features a sharp increase in N_2_ uptake at low relative pressures, indicating the micropores filling, followed by a plateau region. This behavior confirms the presence of a microporous substance with slit-shaped pores. The S_BET_ value of the prepared Cu_3_(BTC)_2_ was 1884.7 m^2^ g^−1^. Additionally, the V_P_ of 0.771 cm^3^ g^−1^ and the average pore width (D_P_) of 1.67 nm further corroborate the microporous nature of the prepared Cu_3_(BTC)_2_, as shown in Figure 2c.

**Figure 3:**
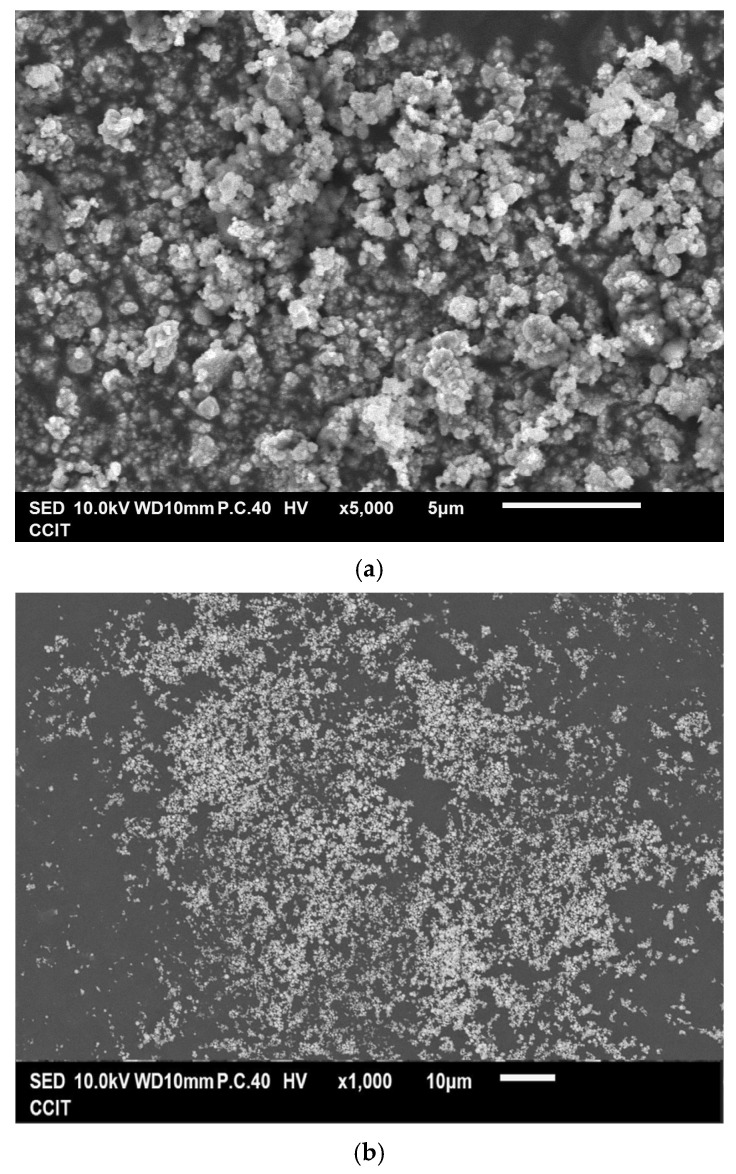

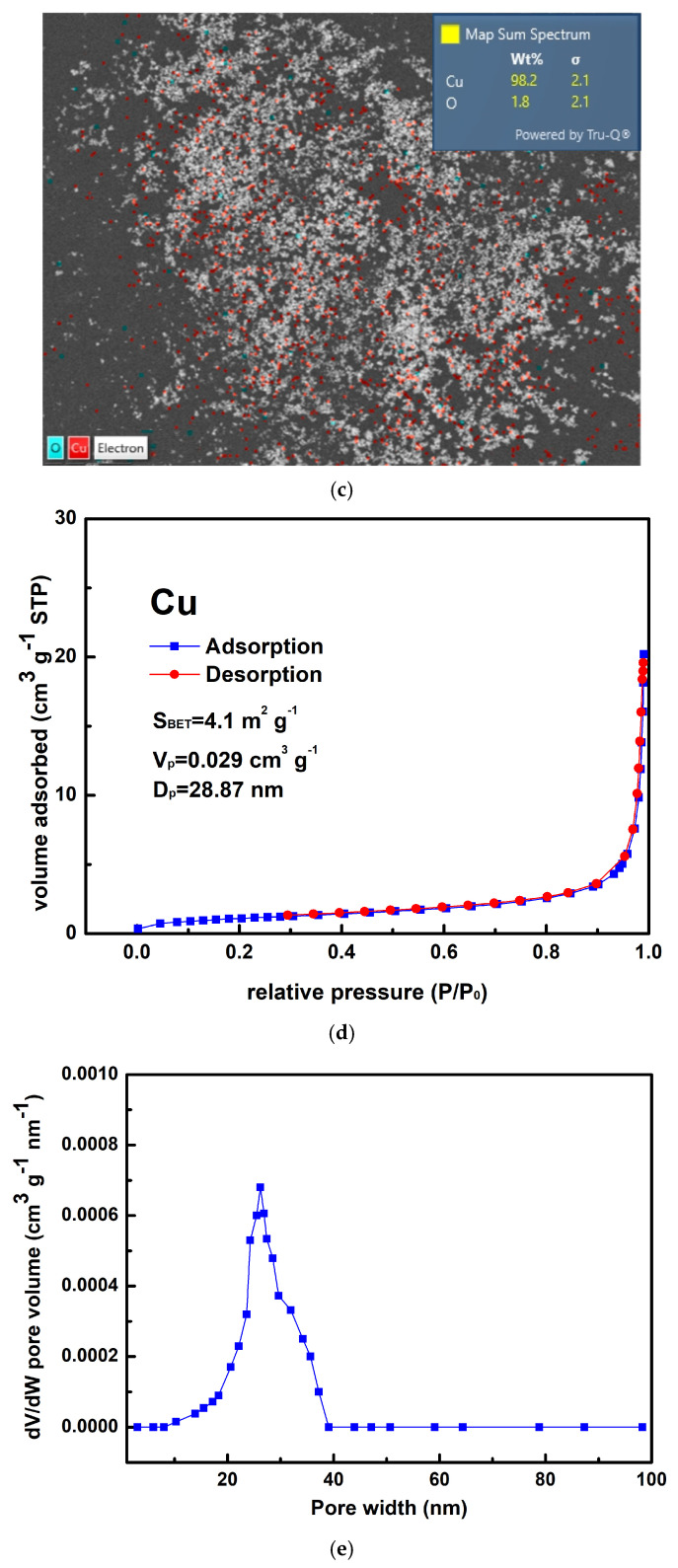
(**a**,**b**) Typical SEM images at different magnifications of the Cu powder, (**c**) the corresponding EDS mapping image, (**d**) the nitrogen adsorption–desorption isotherm of the Cu powder, and (**e**) the pore size distribution curve of the Cu powder.

Figure 3a,b show that the as-prepared Cu powder primarily consisted of agglomerated particles ranging in size from 100 nm to 200 nm. This method yielded 63% for the Cu powder, which was determined by the concentration of Cu^2+^ ions in the raw material. Figure 3c shows the result of the EDS analysis conducted on the prepared Cu powder, revealing the prominent presence of copper (98.2 wt%), along with only a minimal amount of oxygen (1.8 wt%). It is considered that copper readily undergoes oxidation, resulting in the formation of surface oxides; however, these oxides remain undetectable by XRD, as indicated in Figure 1b. Figure 3d presents the isotherm of the sample, categorized as Type III according to the IUPAC classification, characterized by a gradual initial increase in adsorbate uptake, followed by a rapid, exponential increase as the relative pressure rises. Additionally, no hysteresis loop is observed, indicating a reversible process during both adsorption and desorption. The differential pore volume analysis shown in Figure 3e suggests that the agglomerated particles were closely stacked together, with the spaces between them serving as porous structures predominantly composed of micro- and mesopores. The measured values for S_BET_, V_p_, and D_p_ were 4.1 m^2^ g^−1^, 0.029 cm^3^ g^−1^, and 28.87 nm, respectively.

**Figure 4:**
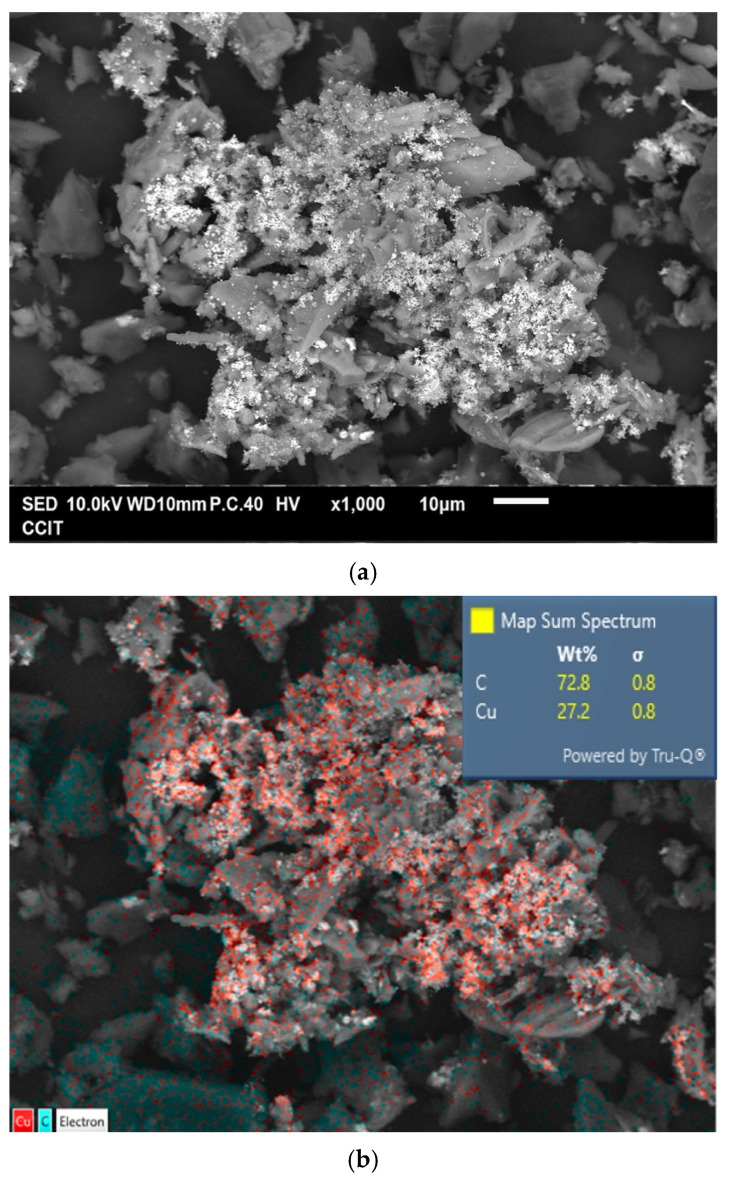

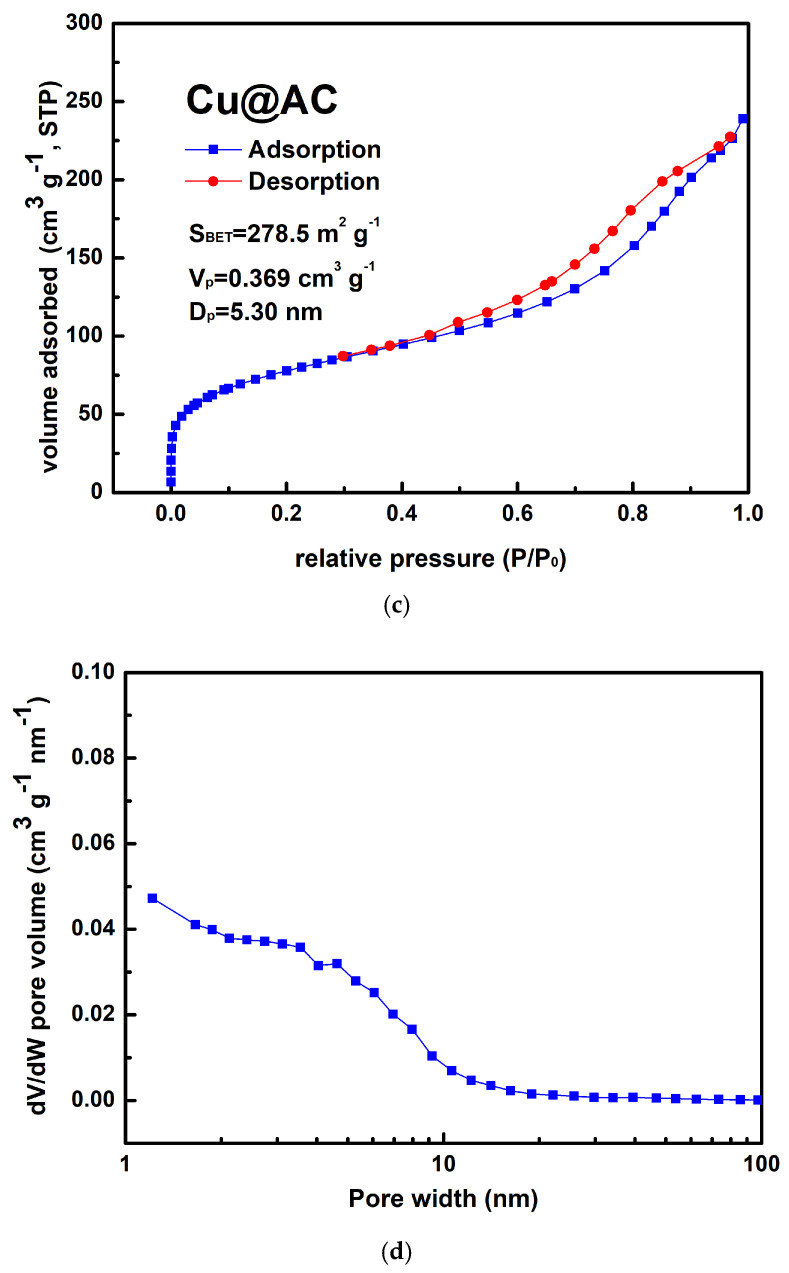
(**a**) Typical SEM image of Cu@AC product, (**b**) corresponding EDS mapping image, (**c**) nitrogen adsorption–desorption isotherms of Cu@AC product, and (**d**) pore size distribution curves of Cu@AC product.

Figure 4a shows the typical morphology of the prepared Cu@AC product. As can be seen, the irregular pieces of AC attached to clusters of aggregated submicron particles. EDS confirmed that these particles were metallic Cu, as illustrated in Figure 4b. The Cu@AC product contained approximately 4.5 wt% Cu, as determined using the EDTA titration method. The adsorption–desorption curve of Cu@AC, shown in Figure 4c, exhibited a Type II isotherm with a H_4_ hysteresis loop, which likely resulted from the microporous characteristics of the AC serving as the support. The SBET of the Cu@AC product was 278.5 m^2^ g^−1^, which was lower than that of the raw AC (~500 m^2^ g^−1^). This reduction was likely due to the deposition of Cu, which could obstruct the pores and interior channels of the AC itself. The V_p_ and D_p_ of the Cu@AC product were measured as 0.369 cm^3^ g^−1^ and 5.30 nm, respectively.

The yield of the three products prepared by the ultrasonic solvothermal approach is listed in Table 1.

### 3.2. Test of Catalytic Effect

**Figure 5:**
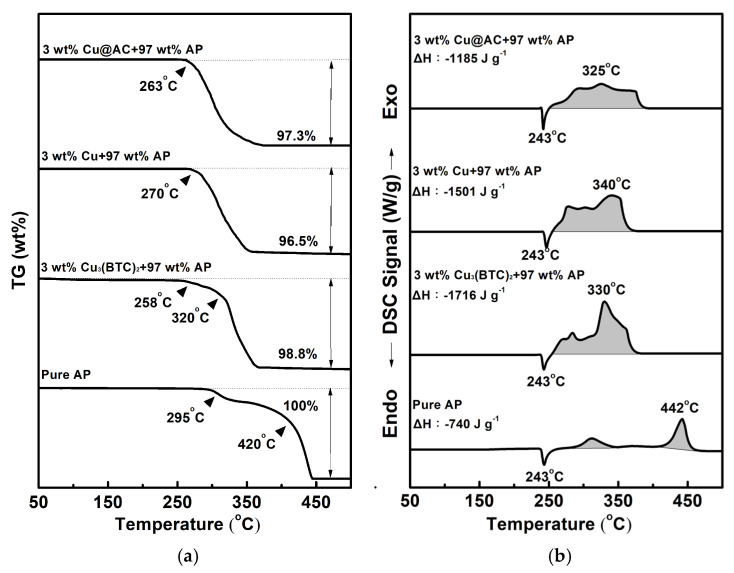
Typical curves of (**a**) TG and (**b**) DSC for pure AP and mixtures containing 3 wt% of various copper-containing substances combined with 97 wt% AP.

Figure 5 shows the typical TG-DSC curves for pure AP and the mixtures containing 3 wt% of various copper-containing substances combined with 97 wt% AP. As shown in Figure 5a, pure AP began to experience significant weight loss at 295 °C, indicating that the thermal decomposition of AP starts at this temperature. The TG curve of pure AP exhibits two thermal weight loss stages: the first occurs between 295 °C and 340 °C (low-temperature decomposition stage, LTD), and the second occurs between 420 °C and 460 °C (high-temperature decomposition stage, HTD). The experimental results are consistent with the literature [4,5,6]. In the mixtures, the initiation temperature for decomposition shifted to a lower value, and the two-stage decomposition was less obvious. In Figure 5b, pure AP exhibits an endothermic peak at 243 °C, indicating a crystalline phase transformation from the orthorhombic to the cubic phase [5,6,7,8]. There are also two exothermic peaks at 312 °C and 442 °C, which represent the LTD and HTD stages of AP, respectively, and the latter is more intense than the former. As for the mixtures, both the LTD and HTD peaks shift to lower temperatures and even overlap; however, the endothermic peak for the crystalline transformation of AP is unaffected. It was found that adding Cu_3_(BTC)_2_, copper powder, and Cu@AC could remarkably reduce the temperature of HTD by 112 °C, 102 °C, and 117 °C, respectively.

As can also be seen, AP underwent complete thermal decomposition within the 25 °C −500 °C temperature range tested, resulting in no residual weight. However, the heat of reaction during the LTD and HTD stages was −740 J g^−1^, which is lower than the theoretical value of −1433 J g^−1^ [5]. This indicates that the most stable gas products may not be generated thoroughly during thermal decomposition. Interestingly, when 3 wt% of the copper-containing substances was present in AP, the amount of heat liberated for the exothermic decomposition of AP increased significantly by 976 J g^−1^, 761 J g^−1^, and 445 J g^−1^ for each substance, and a weight residue of approximately 1.2–3.5 wt% remained. It is speculated that copper-containing substances may react exothermically with AP and can catalyze its thermal decomposition, forming more stable products.

Among the three Cu-containing substances, Cu_3_(BTC)_2_ revealed a notable catalytic effect, reducing thermal decomposition by approximately 112 °C and producing a total heat release two and a half times greater than that of AP. Moreover, to our knowledge, the use of Cu_3_(BTC)_2_ as an additive to foster the thermolysis of AP has rarely been reported. Therefore, we selected Cu_3_(BTC)_2_ to be the focus of the following study.

**Figure 6:**
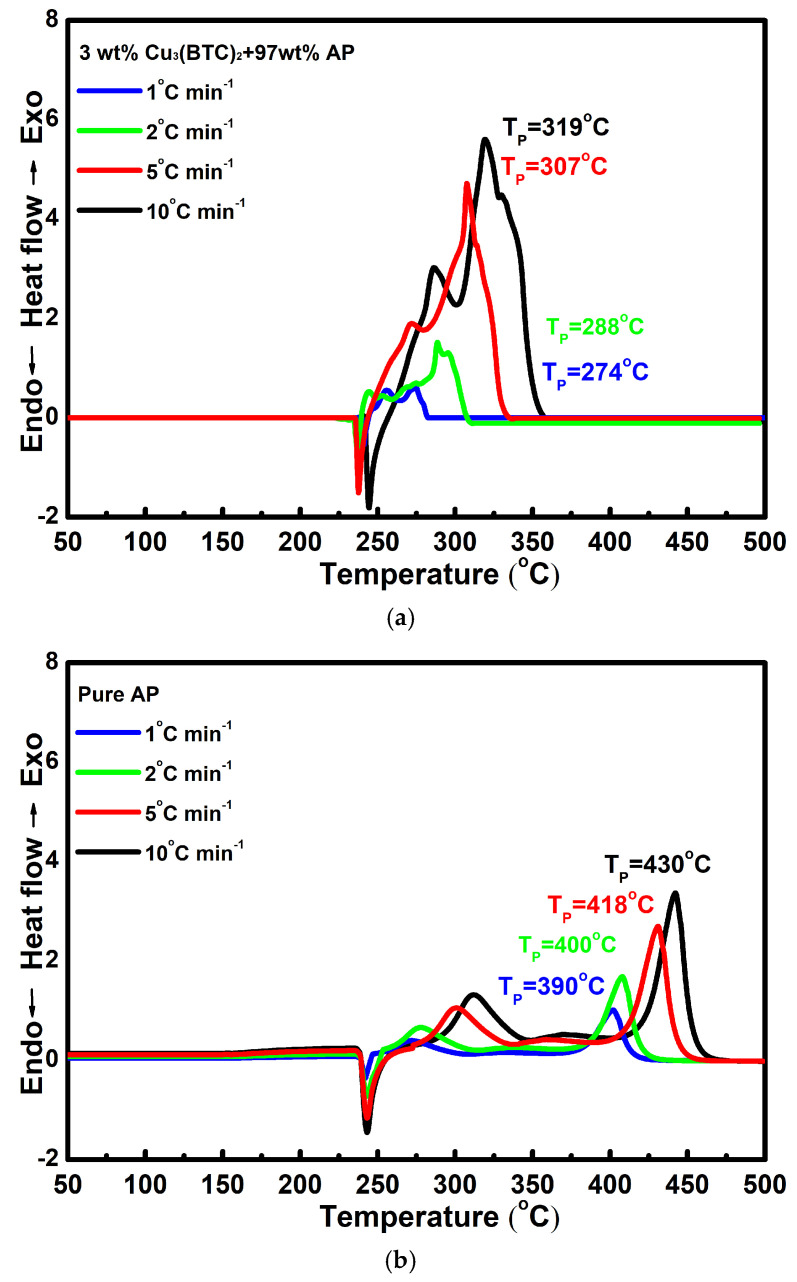
Typical DSC curves for (**a**) the mixture composed of 3 wt% Cu_3_(BTC)_2_ and 97 wt% AP and (**b**) pure AP at various heating rates.

Figure 6a,b show the DSC curves for the mixture composed of 3 wt% Cu_3_(BTC)_2_ and 97 wt% AP and pure AP, respectively, at various heating rates. It can be observed that as the heating rate increases, the DSC curves shift to higher temperatures. This phenomenon can be attributed to the faster heating rate, which created a temperature gradient within the system and led to the uneven heating of the samples. Consequently, the decomposition of AP occurs at a higher temperature when a higher heating rate is applied.

The relationship between the thermal decomposition of energetic materials and the heating rate can be described using Kissinger’s equation [49] and Ozawa’s equation [50], which are expressed as follows:

(1) $$\ln\left(\frac{\beta }{T_{p}^{2}}\right)=\ln\left(\frac{AR}{E_{a}}\right)-\frac{E_{a}}{RT_{p}}$$

(2) $$ln\beta =\left(-\frac{E_{a}}{RT_{p}}\right)+constant$$

where β represents the heating rate, °C min^−1^; T_p_ is the peak temperature (i.e., that at which the AP’s HTD occurred in this study), K; E_a_ is the apparent activation energy, kJ mol^−1^; R is the universal gas constant, 8.314 J (K mol)^−1^; and A is the pre-exponential factor, s^−1^.

**Figure 7:**
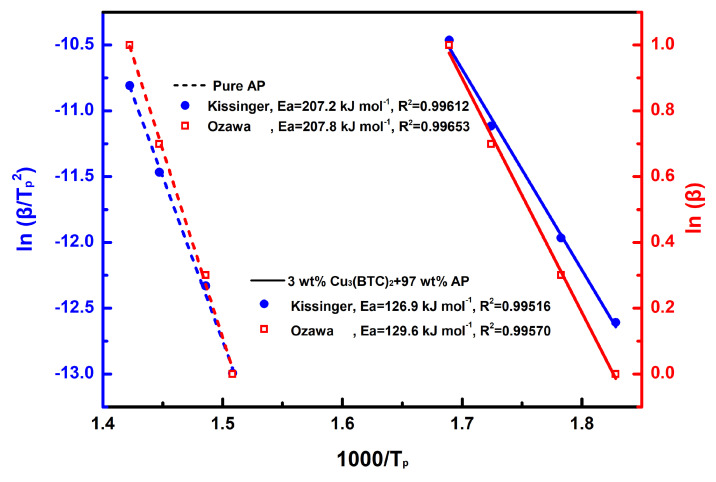
Calculation of E_a_ through a linear-fit relationship using Kissinger’s method [49] and Ozawa’s method [50], respectively, for pure AP and the mixture composed of 3 wt% Cu_3_(BTC)_2_ and 97 wt% AP.

The results of the linear fitting analysis for the 3 wt% Cu_3_(BTC)_2_ + 97 wt% AP mixture and pure AP, utilizing the Kissinger and Ozawa equations, are shown in Figure 7. The straight lines from both analyses show a strong alignment, with a coefficient of determination (R^2^) exceeding 0.995. The E_a_ values determined through the Kissinger and Ozawa methods for the mixture of AP and Cu_3_(BTC)_2_ were 126.9 kJ mol^−1^ and 129.6 kJ mol^−1^, respectively; both were significantly lower than the E_a_ values for pure AP, which were found to be 207.2 kJ mol^−1^ and 207.8 kJ mol^−1^ using the same methodology. This substantial reduction in E_a_ confirms the catalytic effect of Cu_3_(BTC)_2_ in the thermal decomposition of AP.

Table 2 presents the catalytic performance of various MOF additives on the thermal decomposition of AP. Among the MOFs listed in the table, Cu_3_(BTC)_2_ exhibited a substantial and competitive catalytic effect. It significantly reduced the peak temperature of the HTD of AP by 112 °C and decreased E_a_ by 39%. At a concentration of 3 wt%, Cu_3_(BTC)_2_ promoted the thermal decomposition of AP, resulting in the release of heat amounting to 1716 J g^−1^. This heat release can be attributed to the oxidation of the organic group in the BTC ligand and the catalytic effect of Cu^2+^ ions. This interaction enhances the thermal decomposition of AP, leading to a more complete reaction and, consequently, the release of more heat. Further details are provided below.

### 3.3. Thermal Behavior of Cu_3_(BTC)_2_ and Properties of the Resulting Residue After Thermolysis

**Figure 8:**
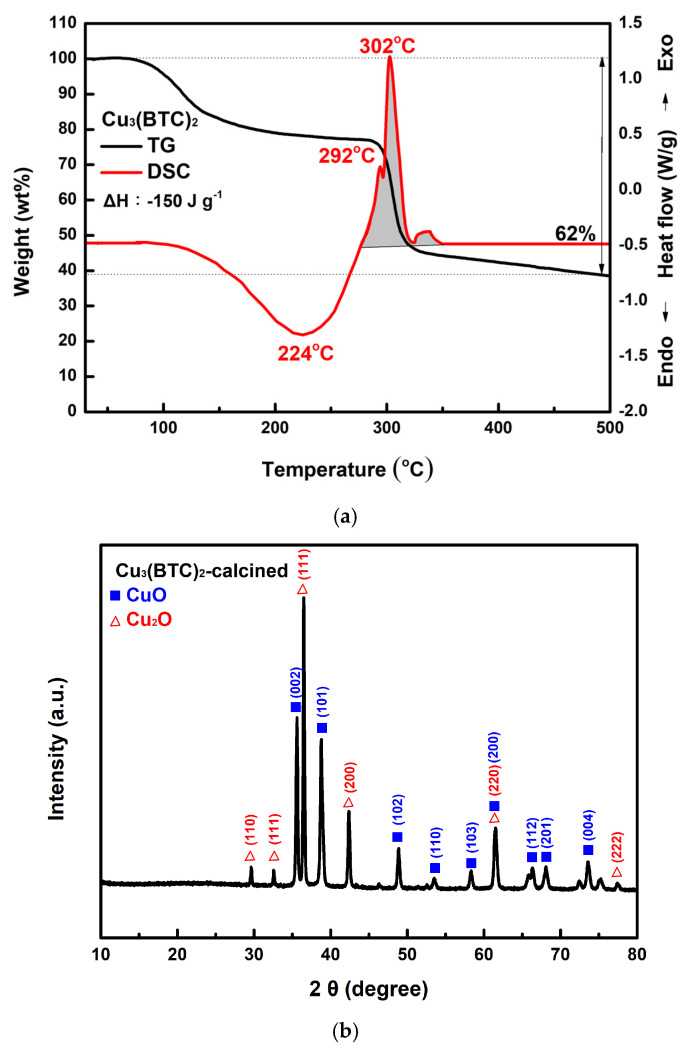

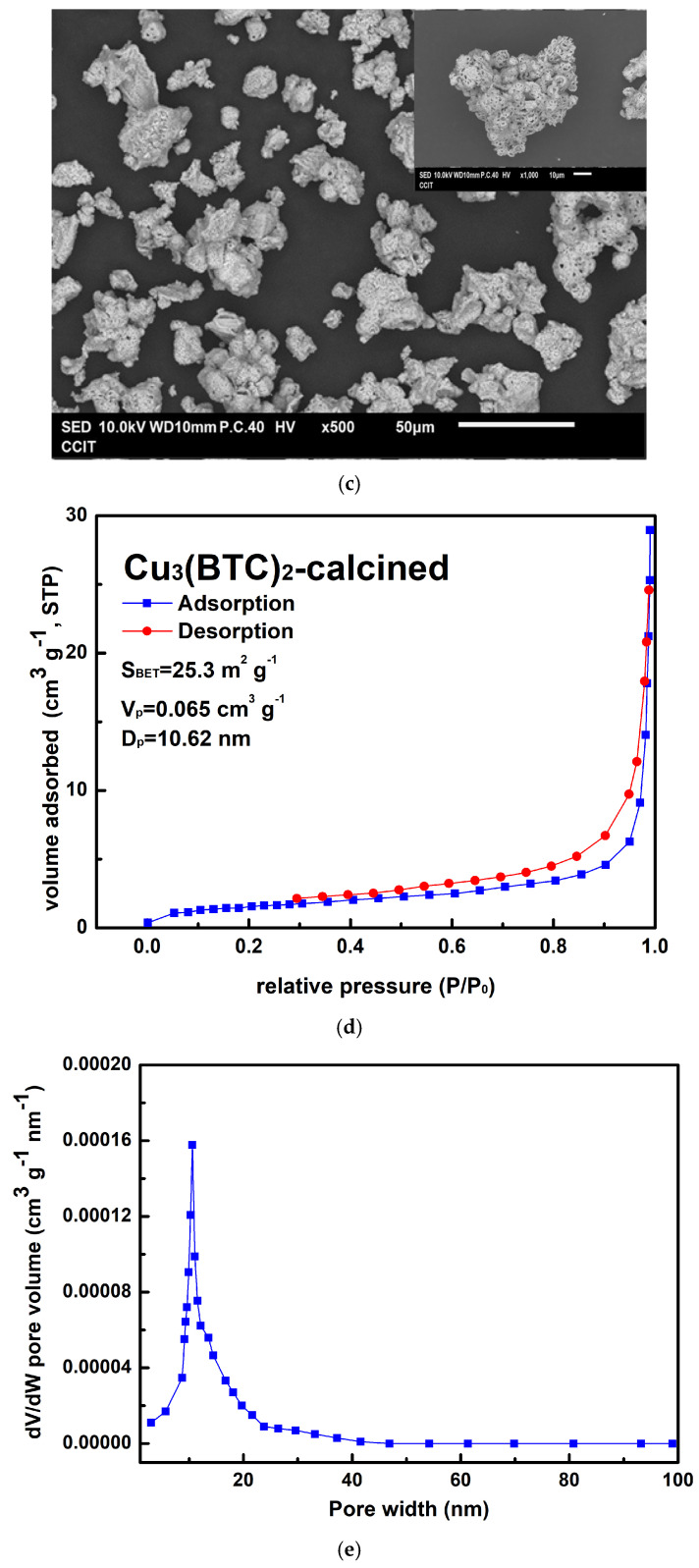
(**a**) Typical TG-DSC curves of the prepared Cu_3_(BTC)_2_, (**b**) typical XRD patterns of the residue after the thermal decomposition of the Cu_3_(BTC)_2_, identified as a CuO/Cu_2_O mixture, (**c**) a typical SEM image of the CuO/Cu_2_O residue, (**d**) the nitrogen adsorption–desorption isotherm of the CuO/Cu_2_O residue, and (**e**) pore size distribution curves of the CuO/Cu_2_O residue.

Figure 8a shows the TG-DSC curves of the prepared Cu_3_(BTC)_2_. The analysis indicates that Cu_3_(BTC)_2_ lost approximately 20% of its weight during dehydration, between 100 °C and 280 °C. Following this dehydration phase, the material decomposed, resulting in an additional ~38% weight loss between 280 °C and 310 °C. The DSC curve exhibits three exothermic peaks after 280 °C. The first two peaks are linked to H_2_O, CO_2_, and Cu_2_O formation. The third peak corresponds to the conversion of Cu_2_O into CuO [32,51]. Figure 8b shows the XRD pattern of the residue obtained after the TG-DSC analysis of the Cu_3_(BTC)_2_. The diffraction peak pattern matches the JCPDS files 45-0937 and 78-2076 for CuO and Cu2O, respectively, indicating the presence of CuO and Cu_2_O phases. According to the Scherrer equation [52], the crystallite size for the CuO/Cu_2_O residue was calculated to be ~33.8 nm.

(3) $$D=\frac{K\lambda }{\beta cos\theta }$$

where D represents the crystallite size (in nm), K is Scherrer’s constant (commonly taken as 0.9), β is the full width at half maximum (in radians), λ is the wavelength of the Cu Kα radiation (0.154 nm), and θ is the Bragg angle (in degrees).

In Figure 8c, the CuO/Cu_2_O residue exhibits a porous, granular structure composed of tiny agglomerated grains. As shown in Figure 8d, the H_3_ hysteresis loop of the CuO/Cu_2_O residue is observed in the Type III isotherm at P/P_0_ values between 0.6 and 1.0. The pore size of the residue of copper oxides was mainly in the region below 20 nm (see Figure 8e). It is thus suggested that micro- and mesopore cracks existed in the sample, which may have been a result of the escape of gaseous species during the thermal decomposition of Cu_3_(BTC)_2_. S_BET_, V_p_, and D_p_ were calculated to be 25.3 m^2^/g, 0.065 cm^3^/g, and 10.62 nm, respectively.

### 3.4. EGA for Thermal Reaction of 3 wt% Cu_3_(BTC)_2_+97 wt% AP

**Figure 9:**
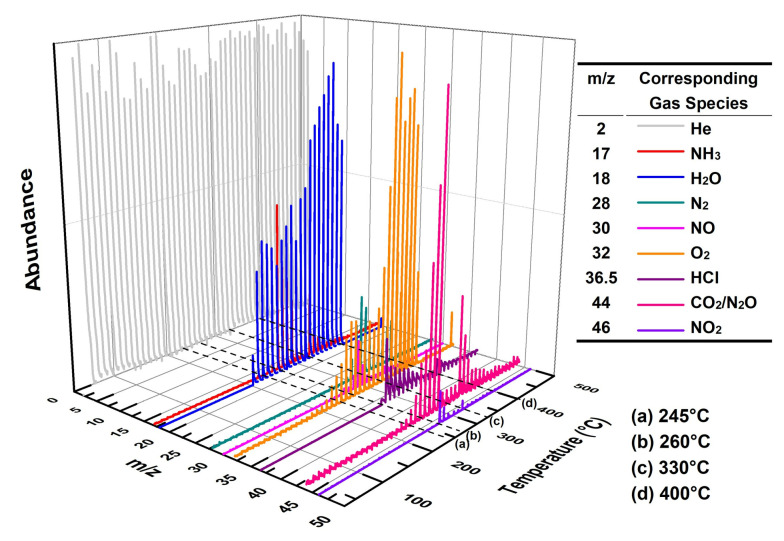
Three-dimensional EGA result for the mixture composed of 3 wt% Cu_3_(BTC)_2_ and 97 wt% AP at a heating rate of 10 °C/min in a helium atmosphere.

Figure 9 shows the three-dimensional EGA spectrum obtained from MS for a mixture of 3 wt% Cu_3_(BTC)_2_ and 97 wt% AP. According to the TG-DSC results for this mixture (refer to Figure 5), four key temperatures are marked in Figure 9: (a) 245 °C, indicating the crystal transformation of AP during the initial heating phase; (b) 260 °C, representing the initiation period of thermolysis; (c) 330 °C, corresponding to the peak temperature during HTD; (d) 400 °C, at which point the residual weight started to stabilize.

At 245 °C, there were minimal emissions of gaseous species, with only trace amounts of H_2_O detected, likely due to the desorption or dehydration of Cu_3_(BTC)_2_. However, at 260 °C, in addition to small quantities of gas products such as NH_3_, N_2_, HCl, and nitrogen oxides (NO, N_2_O, and NO_2_, collectively referred to as NO_x_ hereafter), considerable amounts of H_2_O and CO_2_ were observed. This suggests that Cu_3_(BTC)_2_ participates in the thermolysis of AP. At 330 °C, the signals of these gases became markedly stronger compared to those at 260 °C, indicating a vigorous catalytic reaction between Cu_3_(BTC)_2_ and AP. By 400 °C, the intensities of the gas products diminished, signifying that the reaction was nearing completion.

### 3.5. Possible Catalytic Mechanism

The thermal decomposition of AP is a multifaceted process that involves various chain reactions and intermediate species [5]. It is generally accepted that this decomposition occurs in two stages: LTD and HTD. The rate-determining step in the LTD stage is primarily the formation of NH_3_ and HClO_4_ [53,54]; as for the HTD stage, the rate-determining step may involve the vigorous redox reaction associated with the substantial amounts of NH_3_ and HClO_4_ and the conversion of molecular oxygen (O_2_) into superoxide radical anions (${\cdot O}_{2}^{-}$) [55,56].

**Figure 10:**
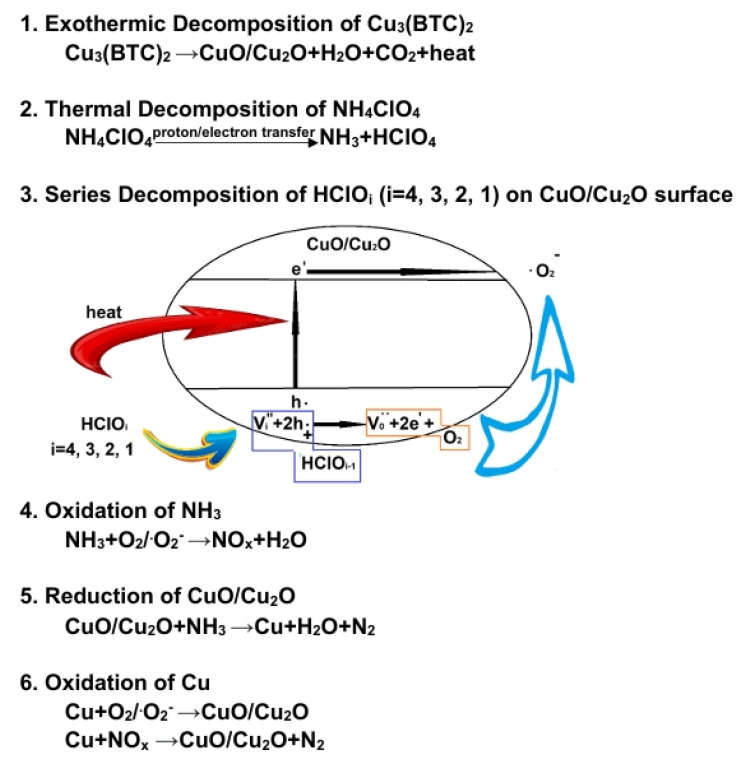
Proposed catalytic reaction mechanism, illustrating AP pyrolysis in the presence of Cu_3_(BTC)_2_.

To understand the possible catalytic mechanism, we can infer the following based on the results from TG-DSC and EGA (see Figure 5 and Figure 9), as illustrated in Figure 10:

NH_4_ClO_4_ decomposes into NH_3_ and HClO_4_ during the thermal decomposition of AP upon heating, which may occur through the electron transfer from ${ClO}_{4}^{-}$ to ${NH}_{4}^{+}$, expressed as follows [31,54]:

(4) $${NH}_{4}{ClO}_{4\left(s\right)}↔{NH}_{4}^{+}+{ClO}_{4}^{-}↔{NH}_{4}^{0}+{ClO}_{4}^{0}↔{NH}_{3\left(ads\right)}+{HClO}_{4\left(ads\right)}$$

Alternatively, decomposition can occur through proton transfer due to N-H bond cleavage [5,57], as shown below:

(5) $${NH}_{4}{ClO}_{4(s)}↔{NH}_{3}-H-{ClO}_{4}↔{NH}_{4}^{0}+{ClO}_{4}^{0}↔{NH}_{3(ads)}+{HClO}_{4(ads)}$$

At relatively low temperatures, the products are adsorbed onto the surface of NH_4_ClO_4_, inhibiting further AP decomposition. Therefore, MS detected no gas produced from AP decomposition at 244 °C; only trace amounts of H_2_O were observed due to the sample’s dehydration/desorption (see Figure 9).

Based on the results shown in Figure 8a, it could be reasonably expected that the mixture containing 3 wt% Cu_3_(BTC)_2_ and 97 wt% AP would easily initiate an exothermic reaction at temperatures below 280 °C. This is due to the organic groups in Cu_3_(BTC)_2_ reacting with the abundant and strong oxidizing agent AP. This reaction produces CO_2_ and H_2_O while leaving behind residual CuO/Cu_2_O. The heat generated from this reaction may provide energy to break the N-H bond in AP, which lowers its thermolysis temperature and initiates its thermal decomposition. This observation aligns with the proton transfer theory. Furthermore, CO_2_ and H_2_O produced from the thermal decomposition of Cu_3_(BTC)_2_ are detected via MS when there is a significant decrease in the sample’s weight, specifically at 260 °C (see Figure 9). As a result, the exothermic decomposition reactions of Cu_3_(BTC)_2_ and AP may facilitate one another during the initiation phase.

The copper oxides CuO/Cu_2_O, formed through the thermal decomposition of Cu_3_(BTC)_2_, can serve as catalysts in the pyrolysis of AP. This is due to the partially filled 3d orbitals in copper ions, which have varying valence states. These characteristics facilitate electron transfer to enhance the decomposition of AP, as expressed below:

(6) $${Cu}_{2}^{2+}+{2NH}_{4}^{+}\to {2Cu}^{2+}+{2NH}_{4}^{0}$$

(7) $${2Cu}^{2+}+{2ClO}_{4}^{-}\to {2Cu}_{2}^{2+}+{2ClO}_{4}^{0}$$

CuO and Cu_2_O are p-type semiconductors with relatively low energy gaps of ~1.4 eV and ~2.1 eV, respectively [58,59]. They are effective photocatalysts for producing hydrogen through water splitting [60] and degrading organic pollutants in water [61]. It is believed that CuO/Cu_2_O also enable the generation of thermally induced electron–hole pairs [62]. The holes produced can attract HClO_4_, and the oxygen atoms from HClO_4_ are absorbed onto the surface of the copper oxides, resulting in the formation of HClO_3_ and additional holes. Subsequently, oxygen molecules are released from the copper oxides’ surface, generating electrons. This process enhances electron transfer to decompose HClO_4_ and may even produce superoxide radical anions (${\cdot O}_{2}^{-}$). It is postulated that the exothermic decomposition of HClO_4_ [63] could be enhanced through the heterogeneous catalysis of CuO/Cu_2_O. Additionally, the deoxygenation of chlorine oxyacids may occur in a series of steps, ultimately producing HCl and O_2_/${\cdot O}_{2}^{-}$ (see the schematic diagram in Figure 10).

The released O_2_/${\cdot O}_{2}^{-}$ can react exothermically with NH_3_, accelerating the thermal decomposition of AP and forming gaseous species, i.e., NO_x_ and H_2_O. At the same time, NH_3_ can reduce CuO/Cu_2_O to form metallic Cu, N_2_, and H_2_O. The excellent electrical conductivity of metallic Cu facilitates electron transfer, aiding in the pyrolysis of AP. The reduced metallic Cu immediately interacts with O_2_/${\cdot O}_{2}^{-}$ and NO_x_, forming CuO/Cu_2_O and N_2_. This regenerative cycling process between copper oxides and metallic Cu can sustain catalytic activity for the thermal decomposition of AP. Cu_3_(BTC)_2_ has a large specific surface area, which enables the derived copper oxides/metallic Cu composites to have an extensive surface and numerous defects in their particle lattices, providing effective active sites for reactions and thus promoting AP decomposition initiated at a lower temperature (265 °C) than that of pure AP’s LTD (310 °C).

As the temperature increases and transitions into the primary thermal decomposition phase of AP (~330 °C), substantial quantities of reactive gases of NH_3_ and HClO_4_ are desorbed from the surface of the AP. This emission causes intensified thermolysis and more vigorous redox reactions in the gas phase, ultimately forming various final products. The composites of copper oxides and metallic Cu absorb these reactive gaseous species on their surfaces, facilitating reactions in the condensed phase. As a result, the thermal decomposition process becomes more thorough due to the formation of more stable products such as H_2_O, N_2_, and HCl, resulting in greater heat release. The calculated ${∆}_{r}H^{0}$ and ${∆}_{r}G^{0}$ at 260 °C and 400 °C for several representative reactions in the proposed mechanism are listed in Table 3 [64]. The negative values of ${∆}_{r}H^{0}$ and ${∆}_{r}G^{0}$ for all reactions indicate that they are exothermic and spontaneous.

As discussed above, the BTC ligand in Cu_3_(BTC)_2_ undergoes exothermic decomposition during the initial phase of the thermolysis of AP. This process produces gaseous CO_2_ and H_2_O, leaving behind a residue of Cu_2_O/CuO. The heat released during this reaction facilitates the AP’s pyrolysis through proton transfer, leading to the generation of HClO_4_ and NH_3_, which subsequently desorb from the surface of AP. Additionally, the heat promotes CuO/Cu_2_O to form electron–hole pairs, aiding the continuous deoxygenation decomposition of chlorine-containing oxyacids and producing O_2_/${\cdot O}_{2}^{-}$. Meanwhile, CuO/Cu_2_O can react with NH3 and be reduced to metallic copper, which possesses excellent conductivity. This metallic copper enhances the catalysis of AP’s pyrolysis via electron transfer. Moreover, metallic copper can react with oxidizing gases, such as O_2_/${\cdot O}_{2}^{-}$ and NO_x_, to regenerate CuO/Cu_2_O. This cycling between CuO/Cu_2_O and metallic copper helps maintain catalytic activity for the ongoing reaction. Thus, the Cu^2+^ ion in Cu_3_(BTC)_2_ plays a crucial role in catalyzing the thermal decomposition of AP.

**Figure 11:**
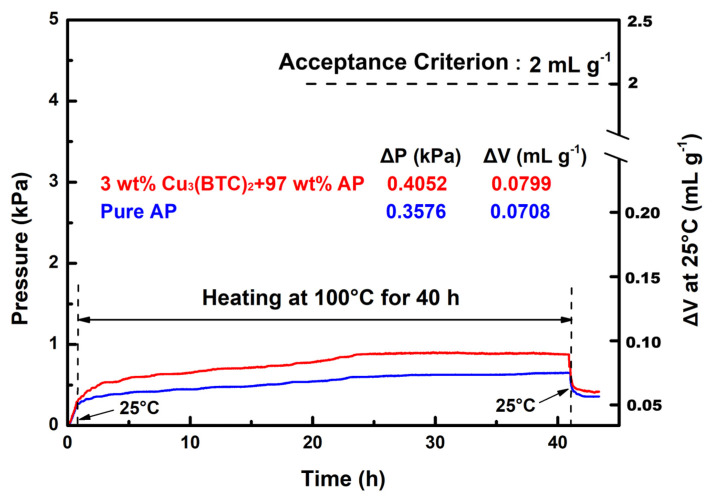
The typical XRD pattern of the prepared Cu powders after contact with AP.

In our experiment, we mixed the prepared copper powder with AP and then dissolved the AP in deionized water. The copper powder was isolated through centrifugation. Following this process, the sample underwent vacuum drying and was analyzed using XRD. As shown in Figure 11, the results reveal the presence of characteristic peaks corresponding to the CuO and Cu_2_O phases, in addition to the distinct peaks of the original Cu.

This finding explains how both the as-prepared fine copper powder and Cu@AC can act as effective catalysts in promoting the thermal decomposition of AP. Once they come into contact, the copper can interact with the strong oxidizing agent AP, leading to partial oxidation and the formation of copper oxides. As described in the proposed reaction mechanism, the as-formed copper oxides subsequently exert a catalytic effect in the thermolysis of AP.

Compared to Cu powder and Cu@AC, Cu_3_(BTC)_2_ demonstrates the most effective catalytic performance in the thermal decomposition of AP. This enhanced performance is attributed to its exothermic decomposition occurring in the initial stages of the reaction, together with the large specific surface area and the predominance of mesopores in the resulting porous copper oxides.

### 3.6. Compatibility Between AP and Cu_3_BTC_2_

**Figure 12:**
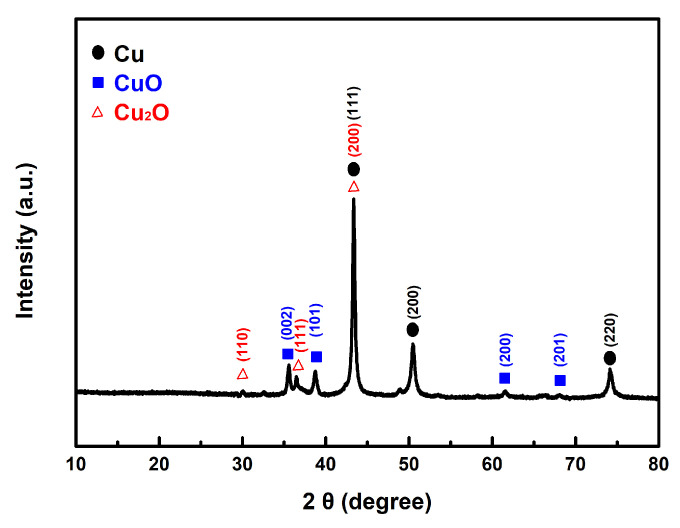
Typical VST curves for the mixture composed of 3 wt% Cu_3_(BTC)_2_ and 97 wt% AP and pure AP.

The VST was employed to evaluate the compatibility of Cu_3_(BTC)_2_ with AP. According to MIL STD-1751A, the gas released by the test sample—comprising 3 wt% Cu_3_(BTC)_2_ and 97 wt% AP—should not exceed 2 mL/g after heating at 100 °C for 40 h. The pressure–time curves for the test sample and pure AP are shown in Figure 12 for comparison.

The results demonstrate that the gas released by the test sample was approximately 0.080 mL/g, which is well below the 2 mL/g threshold established by the acceptance criterion. In contrast, the gas released by pure AP under identical conditions measured ~0.071 mL/g. The pressure–time curves for both substances exhibit a close alignment, indicating a similar trend to that seen with heating at 100 °C during the 1–40 h period, with minimal discrepancies between them. These findings suggest that Cu_3_(BTC)_2_ does not adversely affect the stability of AP and is indeed compatible. Furthermore, in contrast with pure AP, the test sample exhibited a rapid increase in pressure during the initial heating phase, followed by a swift decrease in pressure during the cooling phase. This behavior is believed to result from the dehydration and condensation of hydrated water and the desorption and re-adsorption of gas within the Cu_3_(BTC)_2_ structure.

In this preliminary study, we utilized an ultrasound-assisted solvothermal method to successfully prepare three copper-containing substances that could catalyze the thermal decomposition of ammonium perchlorate, along with a possible catalytic mechanism. The effects of process parameters (such as the reactant concentration, ultrasound power, reaction temperature, and reaction time) on product quality (yield and morphology), as well as the incorporation of these substances into solid composite propellant formulations for the testing and evaluation of burning characteristics, merit further investigation, which is currently underway.

## 4. Conclusions

(1) A one-pot, ultrasonic-assisted solvothermal method was successfully employed to prepare three copper-containing substances: Cu_3_(BTC)_2_, fine copper powder, and Cu@AC. These materials exhibited significant catalytic effects on the thermal decomposition of AP, reducing the initiation temperature and enhancing heat release.
(2) The observed catalytic activity can be attributed to these substances’ appropriate specific surface area and porous nature, which provide numerous active sites for redox reactions. The heat generated from the interactions between the copper-containing substances and AP facilitates proton transfer. Furthermore, the resulting composites of metallic copper and copper oxides serve as effective media for electron transfer. These characteristics collectively promote AP’s thermal decomposition into reactive gaseous species. Notably, the regeneration of metallic copper and copper oxides ensures the maintenance of catalytic efficiency during the pyrolysis of AP.
(3) Cu_3_(BTC)_2_ demonstrated the most effective catalytic performance among the three substances evaluated. At a concentration of 3 wt%, it reduced the thermal decomposition temperature of AP by approximately 112 °C and significantly increased heat release by a factor of ~2.5. The Ea for the thermal decomposition reaction was approximately 128 kJ mol^−1^, approximately 80 kJ mol^−1^ lower than that of pure AP. This standardized VST confirmed the favorable compatibility between AP and Cu_3_(BTC)_2_. These findings suggest that Cu_3_(BTC)_2_ has considerable potential as an additive for AP-based high-energy materials.

## Figures and Tables

**Table 1:** The product yield in this study.

Product	Reactant Cu(NO_3_)_2_·3H_2_O Used	Theoretical Production Amount/Content	Actual Amount/Content Obtained *	Yield
Cu_3_(BTC)_2_	1.812 g	1.512 g	1.240 ± 0.052 g	82 ± 3%
Cu Powder	2.416 g	0.635 g	0.401 ± 0.033 g	63 ± 5%
Cu@AC	0.242 g	6.0 wt%	4.5 ± 0.3 wt%	75 ± 5%

*: The standard deviation obtained by repeating each preparation operation three times.

**Table 2:** Catalytic performance of various MOF additives on thermal decomposition of AP.

Additive	Content (wt%)	ΔT_p_ * (°C)	ΔH (J g^−1^)	Percent Decrease in E_a_ (%)	Reference
Cu_3_(BTC)_2_	3	112	1716	39%	This work
ZIF-67	5	90	2282	56%	[22]
Fe-Co-MOF	5	90	1213	15%	[23]
MIL-88B	-	71	1218	24%	[24]

*: ΔT_p_ is the difference in the peak temperature at which the AP’s HTD occurred between pure AP and the additive/AP mixture.

**Table 3:** The changes in enthalpy, ${∆}_{r}H^{0}$, and Gibbs free energy, ${∆}_{r}G^{0}$, at 260 °C and 400 °C for some representative reactions in the proposed mechanism.

Reaction	260 °C		400 °C	
	Δ_r_H^0^(kJ)	Δ_r_G^0^(kJ)	Δ_r_H^0^(kJ)	Δ_r_G^0^(kJ)
I. Oxidation of NH_3_				
2NH_3(g)_ + 2O_2(g)_ → N_2_O_(g)_ + 3H_2_O_(g)_	−535.90	−531.19	−525.91	−519.96
4NH_3(g)_ + 5O_2(g)_ → 4NO_(g)_ + 6H_2_O_(g)_	−859.94	−955.92	−833.98	−955.22
4NH_3(g)_ + 7O_2(g)_ → 4NO_2(g)_ + 6H_2_O_(g)_	−1085.7	−1025.2	−1055.8	−979.46
II. Reduction of CuO				
3CuO_(s)_ + 2NH_3(g)_ → 3Cu_(s)_ + N_2(g)_ + 3H_2_O_(g)_	−334.54	−367.31	−327.85	−428.62
III. Oxidation of Cu				
2Cu_(s)_ + O_2(g)_ → 2CuO_(s)_	−294.78	−196.02	−282.92	−158.22
Cu_(s)_ + N_2_O_(g)_ → CuO_(s)_ + N_2(g)_	−238.04	−228.16	−237.54	−225.10
2Cu_(s)_ + 2NO_(g)_ → 2CuO_(s)_ + N_2(g)_	−491.36	−379.40	−487.92	−346.56
4Cu_(s)_ + 2NO_2(g)_ → 4CuO_(s)_ + N_2(g)_	−673.26	−540.78	−659.94	−492.66

## Data Availability

The original contributions presented in this study are included in this article. Further inquiries can be directed to the corresponding author.

## Supplementary Materials

The following supporting information can be downloaded at: https://www.mdpi.com/article/10.3390/ma18132928/s1. Figure S1: A flowchart of the preparation of Cu_3_(BTC)_2_ via an ultrasonic-assisted solvothermal method. The process includes the coordination of copper nitrate with trimesic acid in a mixed solvent system under ultrasonic irradiation and subsequent aging at 120 °C. Figure S2: A flowchart illustrating the synthesis of Cu powder. The preparation involves the chemical reduction of copper ions using ascorbic acid in the presence of PVP, followed by filtration and drying. Figure S3: A flowchart showing the preparation of Cu@AC, which includes the impregnation of activated carbon with copper nitrate solution, ultrasonic-assisted reduction, and thermal treatment to obtain the Cu-metalized composite material. All processes were carried out under mild conditions and were optimized to enhance dispersion, uniformity, and safety in handling for AP composite applications.
