# Supplementary material for: Preparation of Cu-Containing Substances via an Ultrasonic-Assisted Solvothermal Approach and Their Catalytic Effects on the Thermal Decomposition of Ammonium Perchlorate
Source: Materials (Basel). 2025 Jun 20;18(13):2928. doi: 10.3390/ma18132928 (PMC12251125; doi:10.3390/ma18132928)
Supplement: Supplementary file 1 [file materials-18-02928-s001.zip › materials-3685088-supplementary.pdf]

Supplementary Materials

Section S1. Flowcharts of Sample Preparation

The following flowcharts illustrate the preparation processes of the synthesized copper-based substances used in this study.

Figure S1. Schematic flowchart for the preparation of $Cu_3(BTC)_2$.

Figure S2. Schematic flowchart for the preparation of Cu Powder.

Figure S3. Schematic flowchart for the preparation of Cu@AC.

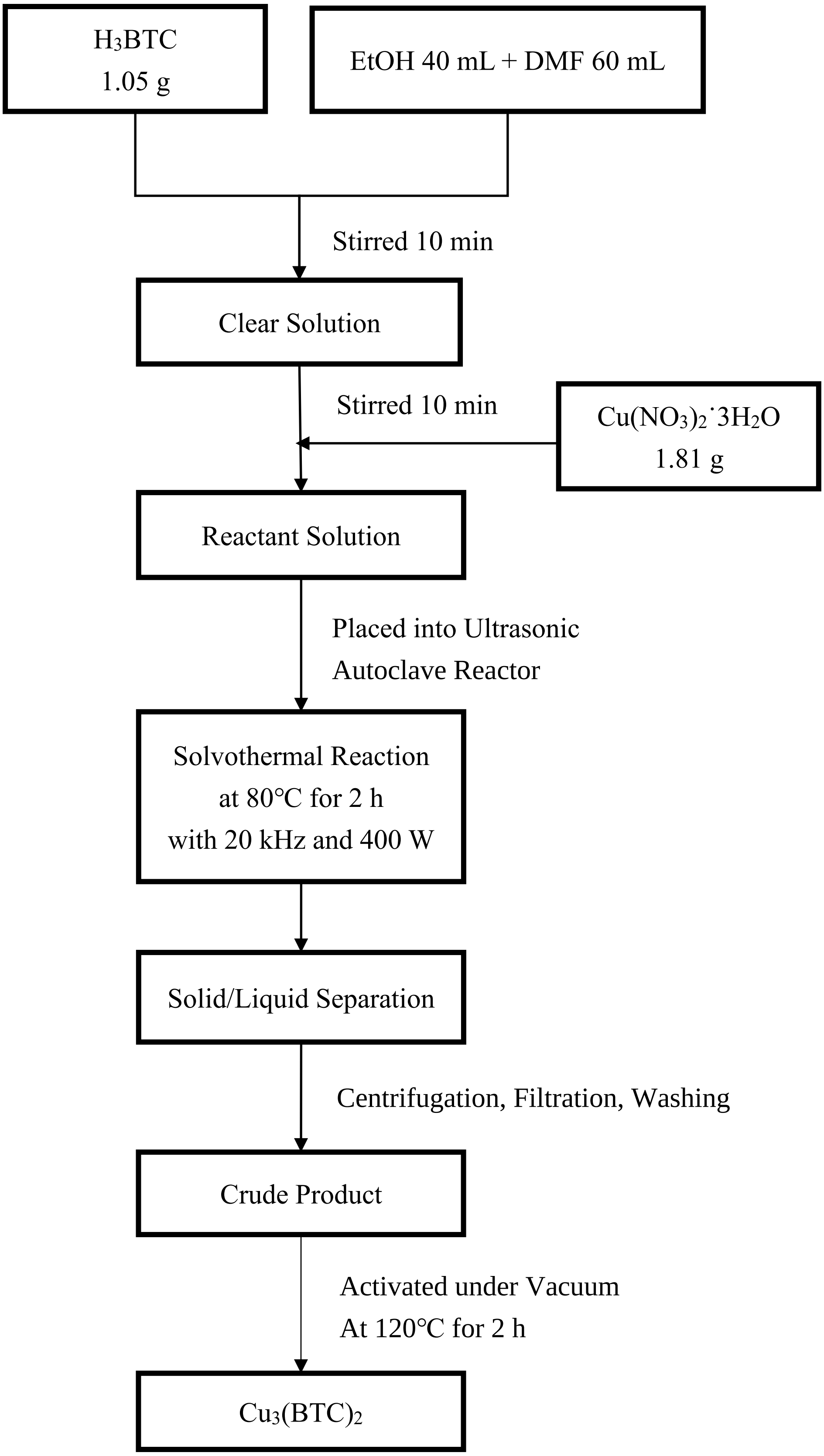

Figure S1. Flowchart of preparation of $Cu_3(BTC)_2$

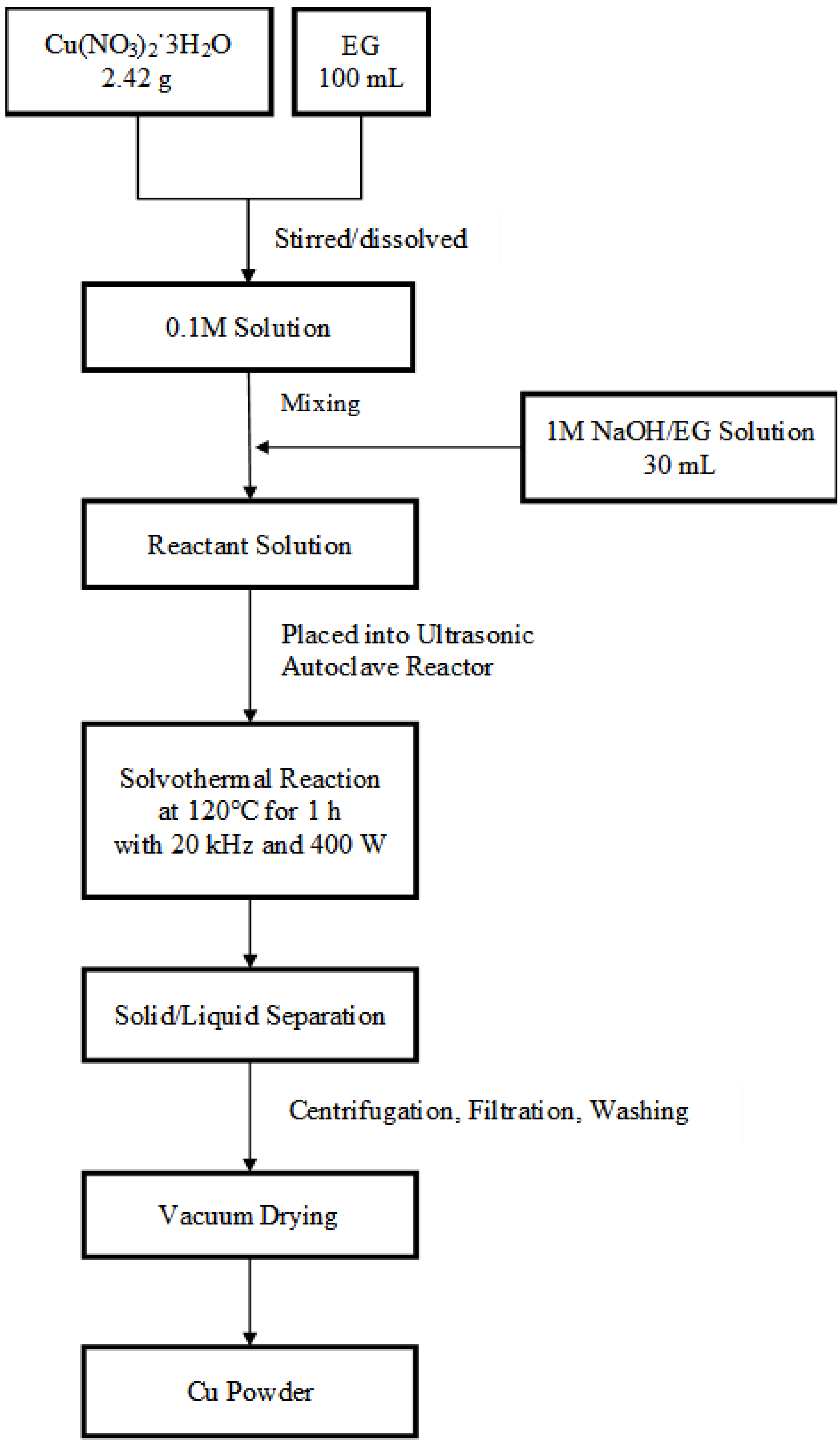

Figure S2. Flowchart of preparation of Cu Powder

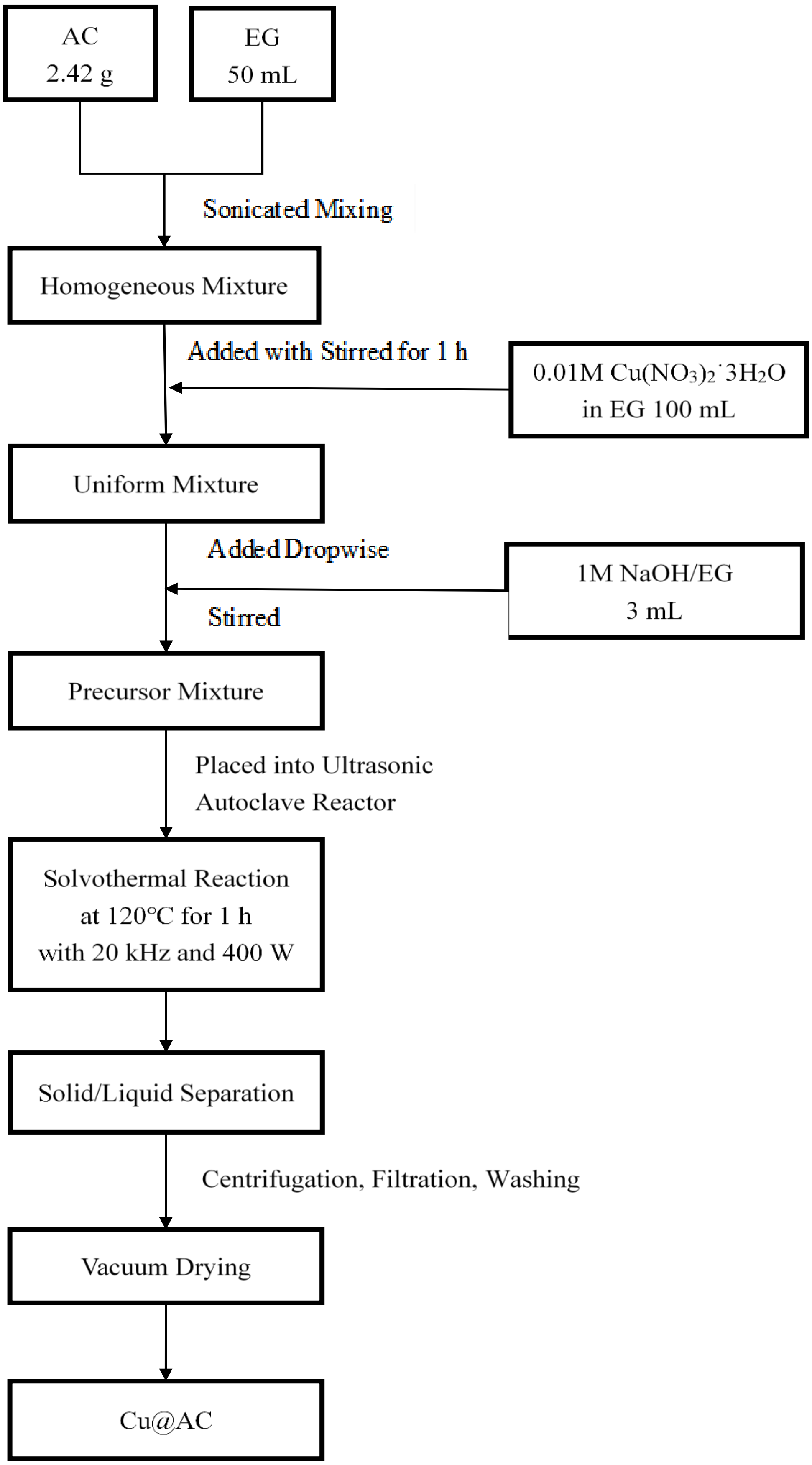

Figure S3. Flowchart of preparation of Cu@AC
